# Oleothorax: A Historic Chest Radiograph Finding

**DOI:** 10.7759/cureus.33305

**Published:** 2023-01-03

**Authors:** Margarida Pimentel Nunes, Inês Branco Carvalho, Isabel Araújo, Raquel Almeida

**Affiliations:** 1 Internal Medicine Department, Hospital Beatriz Ângelo, Loures, PRT

**Keywords:** mycobacterium tuberculosis complex, radiographic interpretation, chest x ray, elderly, lung mass, tuberculosis, oleothorax

## Abstract

Even though tuberculosis (TB) is an extremely old disease, proper effective treatment for the condition became available only around 1944, with the discovery of streptomycin's effect on *Mycobacterium tuberculosis*. Until then, surgical approaches had been among some of the treatments employed, which were dropped with the progressive development of antimycobacterial agents. We present a case of an 83-year-old woman, with a history of pulmonary tuberculosis (PT) at the age of 15 years, presenting with a seven-day history of cough, dark sputum, dyspnea, and pleuritic chest pain. She was submitted to a chest radiograph. The exam revealed a large oval calcified mass on the left apex, compatible with oleothorax. Oleothorax should be included in the differential diagnosis of large calcified thoracic masses in older patients.

## Introduction

Tuberculosis (TB) is an ancient disease, and its origins have been traced back to ancient mummies presenting with typical skeletal deformations of Pott’s lesions in 2400 BC [1], and possibly even before that. However, proper antibiotic treatment was not available for the condition until 1944, when streptomycin’s efficacy in treating *Mycobacterium tuberculosis* was discovered [2]. Until then, treatment had relied on iatrogenic lung collapse, which induced lung cavity closure, and had been only partially effective [3]. This case presents a living example of a historic procedure, performed as a treatment for lung TB in the pre-antibiotic era.

## Case presentation

An 83-year-old woman presented to the emergency department with a seven-day history of productive cough with dark sputum, dyspnea on exertion, symmetrical leg swelling, and left pleuritic chest pain. She had a history of pulmonary tuberculosis (PT) at the age of 15 years, and at that time, she had undergone several procedures that she could not recall. She had not been treated with antimycobacterial antibiotics. She also had hypertension and an ischemic stroke with residual memory deficits. She had recently been submitted to an echocardiogram, which had shown a non-dilated left ventricle, septum hypertrophy, and good systolic function, with no valvular dysfunction. On physical examination, her oxygen saturation was 95% on room air and her heart rate was 105 beats per minute. Auscultation revealed diminished lung sounds on the left posterior hemithorax, where a long thoracic scar was observed. Fine crackles could also be heard throughout the lungs, and mild pitting edema was present on both feet. Her blood workup was relevant for elevated N-terminal pro-B-type natriuretic peptide (NT-proBNP) of 839 pg/mL (normal value: <125 pg/mL) [4], and slightly elevated reactive C-reactive protein of 6.3 mg/dL. No changes in blood count, or kidney or liver function were present (Table 1).

**Table 1:** Patient's blood workup results AST: aspartate aminotransferase; ALT: alanine transaminase; NT-proBNP: N-terminal pro-B-type natriuretic peptide

Parameter	Patient value	Normal range
Hemoglobin (g/dL)	11.9	12-16
Leucocyte (/uL)	9.37	4-10 x 10
Neutrophils (/uL)	6.18	2-7 x 10
Lymphocytes (/uL)	1.79	1-3 x 10
Eosinophils (/uL)	0.31	<0.5 x 10
Platelets (/uL)	252	150-400 x10
Creatinine (mg/dL)	0.52	0.5-0.9
Urea (mg/dL)	44	17-49
AST (UI/L)	14	<32
ALT (UI/L)	9	<33
Lactate desidrogenase (UI/L)	176	135-214
NT-proBNP (pg/mL)	839	<125
C-reactive protein (mg/dL)	6.3	<0.5

**Figure 1:**
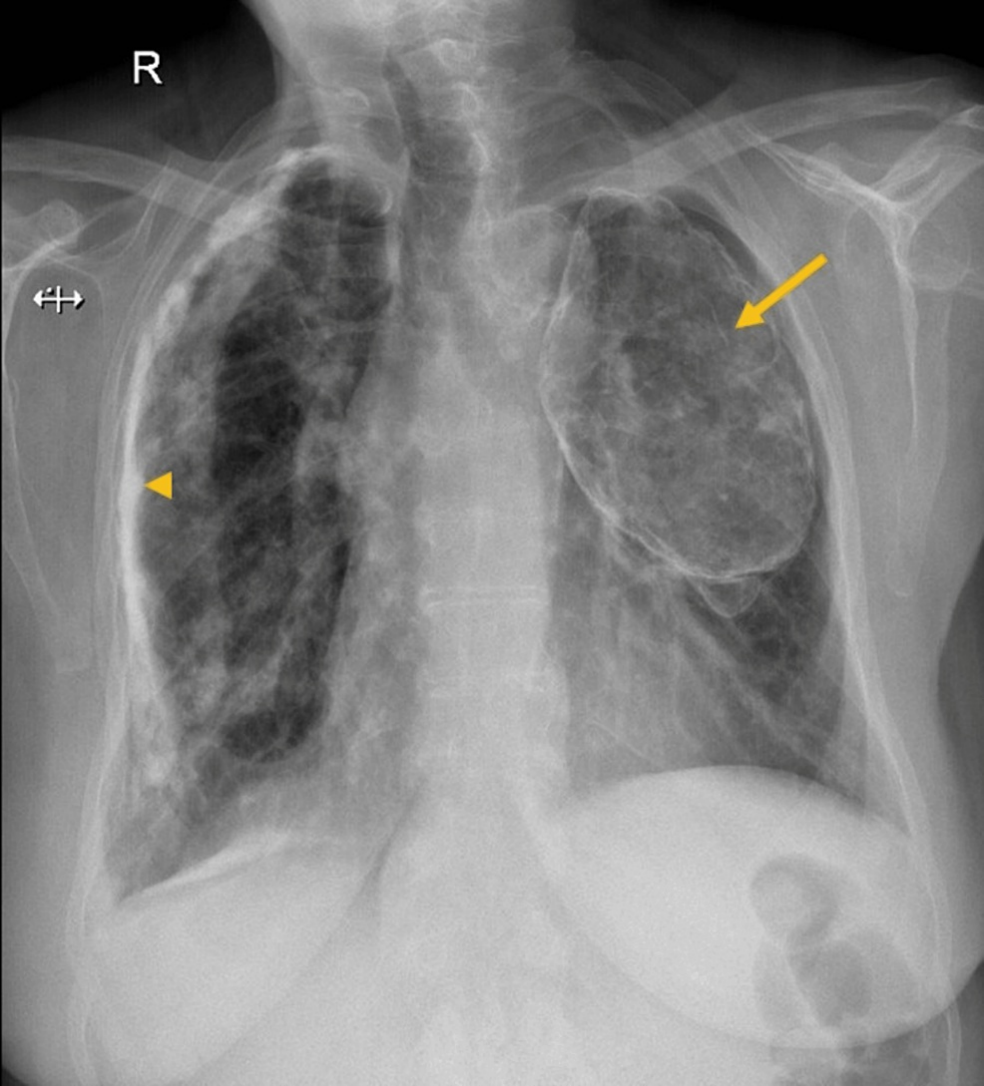
Posteroanterior chest radiograph showing a calcified oleothorax (arrow) and calcified pleural plaques from tuberculosis pleurisy (arrowhead)

**Figure 2:**
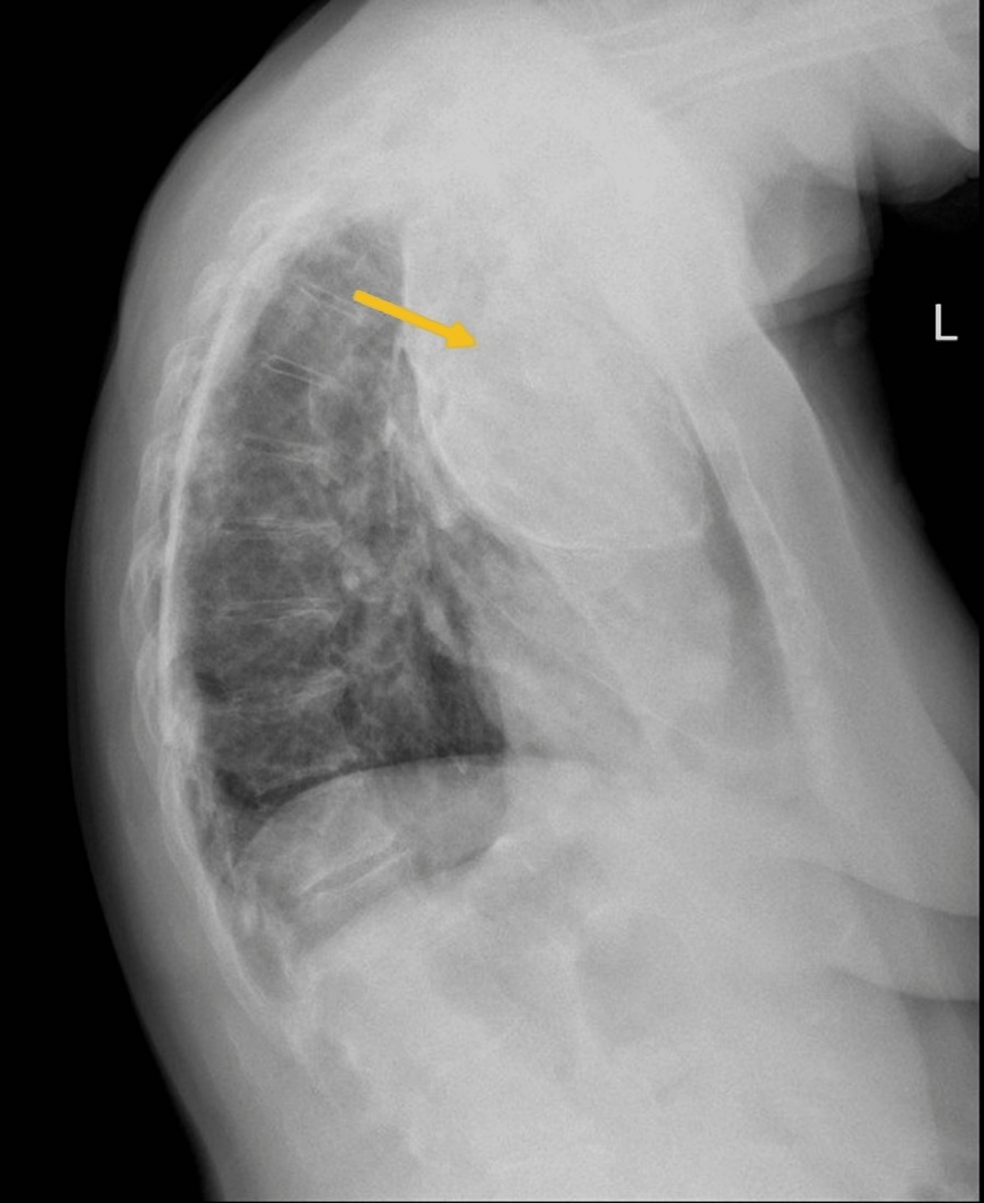
Lateral chest radiograph showing a calcified oleothorax (arrow)

Her chest X-ray revealed an oval-shaped peripherally calcified mass on her left apex compatible with oleothorax (Figures 1, 2), and a linear calcified image (Figure 1) was seen on the internal right lateral thorax wall, consistent with calcified pleural plaques from TB pleurisy. The patient had been previously unaware of this finding and had no previous respiratory or thoracic symptoms.

The patient was diagnosed with *de novo* acute heart failure, triggered by community-acquired bronchitis, and was treated with intravenous furosemide and amoxicillin-clavulanate, and soon discharged with the same medications in oral formulations.

## Discussion

Oleothorax is an obsolete invasive treatment for PT, in which up to 1 liter of an oily substance was temporarily introduced in the pleural cavity, in order to maintain an iatrogenic lung collapse, theoretically inducing an anaerobic and unfavorable environment to the bacilli, by the collapse of the cavities, rendering the patients no longer baciliferous. This was usually performed when a prolonged iatrogenic pneumothorax could not be maintained, due to spontaneous and fast air reabsorption, and the oil was meant to be aspirated out once the symptoms improved. Nonetheless, some patients have kept it in situ [5-7]. This may lead to restrictive lung disease, long-term dyspnea, and an increased risk of other lung infections. Cases of TB reactivation inside the oleothorax have been described in the literature [8].

## Conclusions

Oleothorax is a fading clinical and radiographic finding, which may fall into obscurity soon, since new and more effective treatments for PT are available. Oleothorax should be considered in the differential diagnosis for large calcified thoracic masses in older patients, together with neoplasms that can present with calcifications, namely lung or bone cancer. It can be relatively asymptomatic, but it can cause dyspnea, due to restrictive lung disease induced by extrinsic compression.
